# The Anatomy of Amorphous, Heterogeneous Catalyst Pellets

**DOI:** 10.3390/ma16083205

**Published:** 2023-04-18

**Authors:** Sean P. Rigby

**Affiliations:** 1 Department of Chemical and Environmental Engineering, Faculty of Engineering, University Park Campus, University of Nottingham, Nottingham NG7 2RD, UK; sean.rigby@nottingham.ac.uk; 2 Geo-Energy Research Centre, University Park Campus, University of Nottingham, Nottingham NG7 2RD, UK

**Keywords:** pellet, pore network, porosity, imaging, porosimetry, simulation

## Abstract

This review focuses on disordered, or amorphous, porous heterogeneous catalysts, especially those in the forms of pellets and monoliths. It considers the structural characterisation and representation of the void space of these porous media. It discusses the latest developments in the determination of key void space descriptors, such as porosity, pore size, and tortuosity. In particular, it discusses the contributions that can be made by various imaging modalities in both direct and indirect characterisations and their limitations. The second part of the review considers the various types of representations of the void space of porous catalysts. It was found that these come in three main types, which are dependent on the level of idealisation of the representation and the final purpose of the model. It was found that the limitations on the resolution and field of view for direct imaging methods mean that hybrid methods, combined with indirect porosimetry methods that can bridge the many length scales of structural heterogeneity and provide more statistically representative parameters, deliver the best basis for model construction for understanding mass transport in highly heterogeneous media.

## 1. Introduction

**Figure 1:**
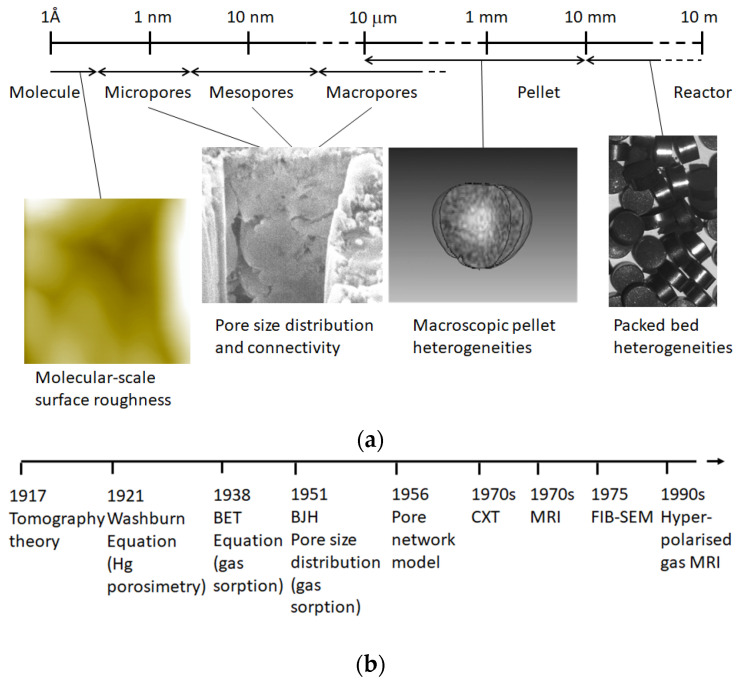
(**a**) Schematic diagram showing the multiscale heterogeneity present in amorphous or disordered porous solids. An example of small-scale roughness in an AFM image of sol-gel silica. An example of pore-scale heterogeneity in a focused ion beam scanning electron microscopy (FIB-SEM) trench for an alumina sample. An example of pellet-scale heterogeneity in a 3D reconstruction of a magnetic resonance image of the porosity distribution (white—high porosity) in a (~3 mm diameter) sol-gel silica sphere. (**b**) Timeline of key inventions in the structural characterisation of porous media, including tomography theory [13], the Washburn [14] equation for mercury porosimetry, Brunauer–Emmett–Teller (BET) theory for gas sorption surface areas [15], the Barrett–Joyner–Halenda (BJH) algorithm for calculating pore size distributions from gas sorption isotherms [16], the pore network model [17], computerised X-ray tomography (CXT) [3,9], magnetic resonance imaging (MRI) [3,18], FIB-SEM [3], and hyperpolarised gas phase MRI [3].

The focus of this review will be on so-called disordered or amorphous heterogeneous catalysts (see Figure 1), rather than crystalline or templated materials, as the former are rather neglected in recent literature but are still industrially important [1,2,3]. The forms of heterogeneous catalysts particularly discussed here are macroscopic pellets and monoliths that are mostly composed of ceramic materials, but other materials are considered when relevant to the broader discussion. Heterogeneous catalyst pellets are typically formed using one of several options, including tabletting, extrusion, and granulation [1,3,4,5]. The feed for the forming step may often have a particle size intermediate between the finished pellet and the catalyst powder raw material [1,3,4,5]. This is because the handling of fine powders is problematic due to issues with static electricity and moisture affecting powder flowability. Feed particle fabrication from powders can involve processes such as spray-drying (SD) and roll-compaction (RC) [3,4,5]. Even if the original powder is agglomerated into an intermediate feed particle, the supplementary addition of a lubricant material, such as graphite, is also required to facilitate the efficient sliding of feed particles past each other in the forming step to ensure efficient consolidation of the particle packing [1,3]. Hence, the physical structure of catalyst pellets has a potential hierarchy that involves levels that arise from the various fabrication stages, including raw powder material synthesis, pelleting feed production, and final pellet formation (as shown in Figure 1).

The heterogeneous catalysts themselves come in a variety of types [1,3]. The active species can form part of the original raw powder material, which is often prepared via precipitation. Alternatively, a full pellet of support material, such as alumina, silica, or carbon, can be formed first, and then the active species is deposited out upon the surface of the said support. In the latter case, the active species typically ultimately consists of nanoscopic crystallites of active metal species dispersed across the support surface. This form for the active species is chosen to provide as large an active surface area for the reaction as possible, with the support aiding the keeping of the metal crystallites from sintering. The active species is often deposited on the support by a process involving impregnation with a solution of the active metal salt, drying to remove the excess liquid, and then calcination. Before the active species may be used in reactions, it may need to be further treated to achieve decomposition of the salt to an oxide and/or reduction of the oxide to metal. All of the aforementioned processing steps, such as drying, calcination, and reduction, may impact the pellet pore structure and thereby influence the final properties of the catalyst [6,7,8,9]. It is shown below how the choices in pellet fabrication can impact mass transport properties.

When the overall activity is diffusion-limited, then the performance of the catalyst depends strongly upon the pore structure [1,10]. Under such conditions, the reactants may only penetrate part way into the pellet before being consumed, and thus, only a fraction of the pellet is usefully employed, which is known as the effectiveness factor [10]. The observed reaction rate, known as the extrinsic rate, differs from that which actually occurs on the active sites of the catalyst, which is known as the intrinsic rate [11]. In most catalysts, the main reaction is still often accompanied by other reactions in series or parallel that cause raw materials and/or target product molecules to be converted into unwanted side products. The relative rates of mass transport of the various side products can impact the final observed selectivity. For example, slowly diffusing molecules are less likely to leave the catalyst pellet before potentially reacting further. Sometimes, the side products can be liquid or solid deposits that occlude the void space of the pellet, further reducing the accessibility and mass transport rates for gaseous reactants [12]. Hence, pore structural characterisation is important for both designing new catalysts and conducting post-mortems on spent catalysts to understand their past performance.

The ‘anatomy’ of the title refers to the structure of the void space of heterogeneous catalytic materials and that of the complementary solid phase, where this is relevant to the nature of the void space over the many length scales seen in Figure 1. This paper reviews the structural characterisation, the contingent description of the void space of disordered porous solids, and the implementation of that description in predicting physico-chemical processes that occur within the void space. Hence, Section 2 considers the key descriptors for characterising the complex structure of disordered porous solids and how these may be obtained. Section 3 includes a detailed discussion of the various ways of representing the structure of the void space of porous media, including both more abstract and image-derived methods. Particular consideration is given to the level of idealisation necessary for different types of porous solids and the physical processes being considered. The focus of the Section 3 is on the relationship of the pore structure with transport processes in particular, rather than its relationship with the catalytic activity, which is an even more complex and multiparametric problem.

The pore structural characterisation techniques covered in this review include more recently introduced imaging methods. In the past, even though tomography theory was invented first [13], as shown in the timeline in Figure 1b, the structural characterisation of porous catalysts was originally dominated by the methods of gas sorption and mercury porosimetry. These techniques are described in detail in several monographs [3,19,20,21,22,23]. More recently, various imaging modalities were progressively introduced. As discussed below, the conventional porosimetries can be used, either in parallel with imaging or as fully integrated methods. Computerised X-ray tomography (CXT) (also called XCT) combines X-ray imaging and tomographical algorithms. It permits the non-invasive study of the internal structure of otherwise opaque materials [3]. CXT involves passing X-rays through the sample via several different paths in multiple directions. This is achieved by rotating the sample within the X-ray chamber so that it can be viewed from different angles. A series of projections (or ‘shadow pictures’ like an old-fashioned medical X-ray) is recorded as the X-ray beam passes through the sample along different trajectories. Once a set of many 2D projections has been obtained, the full 3D image reconstruction is typically achieved using a filtered backprojection algorithm that utilises cone beam reconstruction [3,13]. The reconstruction results in a stack of 2D cross-sectional images, or slices, for the entire sample that represent the best guess by the algorithm for the 3D structure of the sample that would have led to the set of 2D projections obtained. Magnetic resonance imaging (MRI) makes use of the fact that the frequency of precession of the magnetic dipole of the hydrogen nucleus depends upon the strength of the magnetic field it is placed within [3,18]. If a magnetic field gradient is applied to the sample, then the spatial location of a given nucleus can be derived from its precession frequency. Complementary structural modeling approaches were also invented some time ago, in the 1950s, but have subsequently advanced with increasing computing power. Improved computing power has also facilitated data manipulation and analysis for imaging. These developments are discussed in more detail below.

## 2. Void Space Descriptors

### 2.1. Porosity

The porosity, or voidage fraction (ratio of void space volume to overall sample bulk volume), is the key characteristic parameter of a porous solid [3]. However, for disordered solids, the local value can vary extensively across a given pellet. This, often macroscopic, variation in the spatial distribution of porosity was shown to affect mass transport [24,25,26]. Hence, there is a need to map this variability. For systems where all the void space features in the requisite sample volume are above the highest resolution possible with an imaging modality, then this can be done directly [27,28]. Notwithstanding image artefacts, the only issue tends to then be the uncertainty and errors arising from the image segmentation, or gating, procedure that distinguishes the solid phase from the void volume [29]. However, this only affects the boundary layer region of voxels, which, if the resolution is fine compared with the pore sizes, then just represents a small fraction of the whole image volume, and is thus insignificant. However, as the ratio of the pore size to image resolution drops, this segmentation error can become a substantial part of the whole [29].

In addition, for many hierarchical porous solids with void features over many length scales, some of the voids may be below the imaging resolution limit. However, several methods were developed to map heterogeneous spatial distributions of porosity where the void space structure itself is below the imaging resolution limit and/or where the required field-of-view limits the resolution possible.

For CXT, a high-electron-density contrast agent can be added to the void space to enhance the difference in X-ray absorbance between the void and solid phases. A variety of contrast agents were used, including tri-iodomethane [30], xenon gas [31] and mercury [32]. Some contrast agents, such as mercury, have very high density and dominate X-ray absorbance. However, for more precise measurements, some sort of internal reference or calibration procedure is needed to convert the image voxel intensity into a porosity value.

Three-dimensional ptychographic X-ray computed tomography (PXCT) is able to provide 3D maps of the electron density of the mesoporous solid matrix, and thence, can also map the spatial variation of the total (and not just surface accessible) mesoporosity [33]. However, converting the observed electron density into an inferred mesoporosity depends upon the chemical composition of the intrinsic solid matrix being known, along with the assumption that this composition is homogeneous across the sample. Hence, the derivation of mesoporosity values is subject to errors, including those due to the presence of contaminants (as are often left over by many material syntheses), making the composition vary in unpredictable ways. A potential systematic error in the electron density values from measurements made near the sample surface occurs due to artefacts that arise from surface defects. Nevertheless, PXCT was used to show that the calcination of alumina pellets resulted in the densification of a mesoporous matrix and the consequent loss of mesoporosity [33].

Magnetic resonance imaging (MRI) can also be used to provide maps of the macroscopic heterogeneities (>10 μm) in the spatial distribution of porosity [34,35,36] (see Figure 1). Porosity maps can be obtained from NMR relaxation time pre-conditioned imaging sequences that deliver the local NMR spin density in each voxel deconvoluted from relaxation-time-induced contrast effects. The spin density map is initially at an arbitrary scale but can be calibrated against a suitable standard if exact porosity values are required. This method was used to map heterogeneities in porosity introduced by various pellet-forming procedures, such as tabletting, extrusion, and granulation [24,34,35,36,37,38]. However, the presence of significant amounts of paramagnetic impurities (including common species such as iron-III or copper-II) can destroy the NMR signal strength entirely or create systematic errors in the measurement due to their influence on NMR relaxation time rates. However, these impurities are often only in trace amounts uniformly across the pore surface, which does not impact the measurement; furthermore, their influence can be shielded by being covered with thin barrier layers, such as water films for measurements with hyperpolarised xenon gas [39]. However, if the catalytically active site itself is paramagnetic and the support otherwise lacks significant paramagnetics then the drop in NMR signal strength can be actively used as a way to determine the accessibility of the active sites by diffusing gas molecules [39]. If the water barrier is selectively removed from progressively smaller pores, then when the NMR probe molecule finally contacts the paramagnetic species, the NMR signal strength drops noticeably, and the key porosity providing access to the catalytic sites can be determined [39]. Alternatively, measurements with spin-1 nuclei, such as deuterons (in heavy water), are also less susceptible to interference from paramagnetic impurities [39].

Scanning electron microscopy (SEM) operating in backscattered electron (BSE) mode can be used to obtain local porosity measurements for the relevant field of view [40]. This makes use of a ‘mixing model’ to relate the sample atomic composition to the backscattering yield and the filling of the porosity with a resin of known composition. The method was validated against independent measurements of porosity and then used to study the heterogeneities in porosity distribution in porous aluminas resulting from fabrication process conditions, such as kneading energy [40].

### 2.2. Pore Size

While an imaging modality may potentially supply a complete direct visualisation of the void space of the porous solid, the simple visual examination of a 3D rendering of the structure on a computer may not be particularly informative. The form of the void space thus needs to be parameterised with suitable descriptors. In order to make the unwieldy whole void space more tractable for understanding the differences between materials, it is often partitioned into smaller fragments known as pores [3]. Each pore, once identified, might be assigned its characteristic size, and the overall distribution of the incidence of these different pore sizes is a key descriptor of a given porous solid, which can be used to compare it against others. While the definition of an individual pore is straightforward for the completely isolated ‘bubble-like’ pores that are entirely enclosed by solid phase, as in a foam structure, when the void space is more interconnected, the objective definition of an individual pore is more difficult. Dullien [41] proposed that a pore could be defined as a portion of void space that is bounded by a solid surface and planes erected where the hydraulic radius of the void space exhibits local minima.

For a 3D, regular lattice-based rendering of the void space obtained from imaging, a suitable image analysis algorithm can be used to identify the local hydraulic radius minima. One such algorithm is morphological thinning [42]. This consists of removing (or changing the status to a new ‘solid’ phase) void space voxels suitably defined as belonging to the edge of the void or interface with the solid voxels. This procedure can be repeated for successive layers of voxels, thereby thinning down the void volume. The regions that thin early in the process are typically pore necks and those that thin afterwards are the pore bodies. The thinning process thus identifies the pore necks and allows for the partitioning of the void space up into individual pores. However, the image analysis algorithm is an abstract process, and all algorithms do not always give rise to the same resultant partitioning due to variations in the rules of the algorithms, such as whether voxels that only meet at corners rather than share a complete face are considered nearest neighbours.

It is often suggested that the pore size distribution obtained from such a partitioning procedure from an image is likely to be more accurate than a (supposedly) more indirect method, such as gas adsorption. However, the physical processes involved in the multilayer build-up and condensation during gas adsorption are somewhat analogous to the abstract morphological thinning procedure. The adsorbate film tends to grow via the addition of successive layers as the gas pressure is increased, analogous to each generation of interfacial voxels added to the morphological thinning. Further, during adsorption, pore necks tend to fill with condensate before pore bodies, just as necks thin earlier during the application of the thinning algorithm compared with pore bodies. Hence, obtaining pore size distributions from gas adsorption is not so far removed from doing so via morphological thinning of an image of the same void space, as might be supposed. Indeed, recent simulations of gas adsorption using geometrical thinning algorithms were performed and found to be predictive of the form of adsorption isotherms for a range of more ordered and disordered porous solids [43,44]. Further, gas sorption scanning curves can also be used to physically partition the void space into useful subregions [45], the parameters (such as void volume) of which will correlate with zones of particular phase transitions (and thus, characteristic pore size) [46] or mass transport properties [45]. Gas sorption scanning curves are gathered from experiments where the direction of change in the gas pressure is reversed before the top of the adsorption isotherm or lower hysteresis closure point on the desorption branch is reached.

It was mentioned above that the ratio of the pore size to the voxel resolution is the most important factor that determines the limit on the capabilities of a given imaging modality, especially for the ‘direct’ determination of pore sizes. In addition to the removal of image artefacts that might be falsely confused with pores, image segmentation is the key image analysis process that affects the accuracy of pore size measurements via direct imaging, as the method of deciding the boundaries of pores impacts their apparent size. This process remains prone to subjective elements in the various proposed algorithms and associated procedures [29].

In addition, certain imaging modalities can be augmented to provide maps of the larger-scale spatial distribution of pore sizes below the voxel resolution. For example, NMR relaxation time preconditioning can be used to produce spatially resolved maps of the distribution of local average pore size over macroscopic length scales (>10 μm) using MRI [34,36]. These particular types of heterogeneities were seen to influence mass transport rates using complementary MRI studies of transient diffusional liquid–liquid exchange [24].

Even once individual pores are identified in some way, the characteristic size of pores in an amorphous material is ambiguous. Generalised definitions of characteristic sizes include the hydraulic mean diameter defined in terms of the surface-area-to-volume ratio of the pore [41]. However, some apparently amorphous materials can possess pores of a form that closely resembles Euclidean models, such as cylinders or spheres. For example, the width of the hysteresis between melting and freezing curves in cryoporometry can be used to determine the pore geometry [47]. This method showed that the pores of some sol-gel silicas are approximately cylindrical [48].

For cylindrical pores, besides the diameter, another key parameter is the pore length, and thence, for the porous material, the pore length distribution. This is because refinements of the basic theory for the Knudsen diffusion mechanism showed that the Knudsen diffusivity depends on the pore length [2]. For example, when the pore length is the same as the diameter, the Knudsen diffusivity is a factor of three-sevenths different from that predicted using the original Knudsen formula for long cylinders. Further, simulations of adsorption showed that the capillary condensation pressure is also a function of the pore length [49,50]. Indeed, since very long pores were found to behave like dead-end pores for capillary condensation [50], and most macroscopic amorphous materials have long pores, then this suggests that the pore size distribution from the adsorption branch should be obtained with the hemispherical meniscus in the Kelvin equation [3,16]. However, despite the importance of pore length as a parameter that affects key physical processes that arise in catalysis, methods for the determination of pore length distributions are few.

Rigby and co-workers proposed a methodology to obtain pore length distributions that used the particular experimental dataset available from the fully integrated gas sorption and mercury porosimetry method [51] and analysed it with an elaboration of the Seaton [52,53,54,55,56,57] percolation-based analysis by utilising additional concepts from graph theory and probability theory [58,59]. This method supplies the exponent for a power law relation between pore diameter and length, and its potential distribution across pore diameters. However, it is noted that the model still makes use of some of the underlying assumptions of the original percolation analysis, such as a completely random arrangement of pore sizes across a bond network and, therefore, a completely random distribution of entrapped mercury. It also makes similar assumptions about the pattern of mercury entrapment at network nodes as was made for simulations of porosimetry by Portsmouth and Gladden [60,61] that were, themselves, based upon empirical findings from porosimetry experiments on glass micromodels [62].

An alternative method used to obtain pore length is that proposed by Pomonis and co-workers [63,64]. This method uses the incremental surface areas *S_i_* and pore volumes *V_i_* for each pore radius *r_i_* in the pore size distribution to obtain the exponent *α_i_* in a power law relation between the pore length and radius from a plot of

(1) $$log\left[\left(S_{i}^{3}/V_{i}^{2}\right)/\left(16\pi \right)\right]=log\frac{N_{i}}{2}+\left(\alpha_{i}-1\right)logr_{i}.$$

If a pore size distribution is used to obtain the value of *α_i_*, then it is subject to the usual uncertainties in the determination of the pore size distribution, namely, the mode of capillary condensation assumed and errors due to multilayer build-up in macropores if the capillary condensation does not pore fill the material at the top of the isotherm. This method was tested by comparing the pore anisotropy *b_i_*, which is defined as the ratio of the length to the diameter for both fresh (non-functionalised) and functionalised templated silicas. This showed that, as might be expected, the addition of small functional groups led to an increase in *b* as the pore diameter was simply narrowed, while the addition of large groups led to a decrease in *b* as they blocked off pores and thereby shortened the remainder.

The pore length distribution can also be determined from image analysis of full 3D reconstructions of the void space, where the morphology was such that nodes (junctions) in the pore network can be clearly identified, and thus, the endpoints of individual pores are clearly demarcated. This is relatively straightforward where void spaces are reduced to the underlying graph of nodes and branches [65].

### 2.3. Tortuosity Parameter

The tortuosity of a porous catalyst is often used to obtain an effective diffusivity for coupled diffusion and reaction calculations. Effective diffusivity is typically defined as the product of the porosity and reference diffusivity, divided by the tortuosity factor. The nature of the reference diffusivity depends upon whether diffusion is in the molecular or Knudsen regime. Tortuosity is a void space descriptor related to its topology, but it is often conceptually vague and ill-defined [66,67]. This is because parameters obtained via a range of incommensurable methods are all called tortuosity. While tortuosity is related to pore connectivity, it is different because, even though it often purports to be a purely geometric parameter, it typically incorporates a contribution from the boundary conditions of mass transport to which it relates. This is because tortuosity is often defined in relation to mass transport parameters, such as effective diffusivity and permeability. Fu et al. [67] referred to tortuosities based upon transfer processes as ‘physical tortuosities’.

There are also purely geometric definitions of tortuosity that are based upon deviations from the straight-line distance between two points in a void space. However, the particular choice of the pathway within the porous solid is not clear [68]. The tortuosity is often defined as the ratio of the shortest feasible path between two points in the void space and their straight-line distance apart, or as the ratio of the average length of the various potential pathways between the two points and their straight-line separation [66,67]. The latter definition has an issue in that the choice of the weighting of the averaging can be various, including number-weighting of paths or via the typical or minimum cross-sectional area en-route or via a transport flux-weighting. Since the resistance to transport, of a particular pore, depends upon the nature of the diffusion regime, as molecular and Knudsen diffusion have different dependencies on pore size, a flux-weighted average will depend upon the diffusion regime. Morgado Lopes et al. [68] suggested that measurements of tortuosity based upon conductivity, permeability or diffusivity are equivalent to the aforementioned ratio of distances squared.

In any case, tortuosity, like porosity, is a parameter that can be defined for a particular volume of space, but, while not strictly a vector quantity, also potentially incorporates an implicit directional component that arises from the boundary conditions of the mass transfer process to which it refers. The type of mass transfer process studied in a measurement of tortuosity affects the value obtained [3,34,35,36]. For determinations of tortuosity from diffusional measurements, the boundary conditions of the experiment can involve a transient or steady state and have a concentration-gradient-driven diffusional flux or involve steady-state self-diffusion without any externally imposed driving force. Mann and co-workers [69,70,71] and Hollewand and Gladden [72,73] also considered diffusional tortuosity obtained under reaction conditions in diffusion-limited, heterogeneous catalytic processes. The boundary conditions of these experiments mean that the molecular migrations would sample a spatially heterogeneous void space in different ways and to different extents, and thus, the tortuosity thereby obtained would be weighted by different aspects of the void space. A key issue with measurement via steady-state diffusional flux methods (e.g., a Wicke–Kallenbach-type apparatus [74]) is that the tortuosity value is often weighted towards the so-called ‘transport pores’ along which most of the flux transits the material [41,75]. In contrast, in the transient gas uptake and steady-state self-diffusion experiments, the probe molecules will also explore dead-end pores too. Under diffusion-limited reaction conditions, the diffusional flux only samples the outer, mantle region of the porous material, where consumption of the incoming reactant molecule is still occurring. Mann [69,70,71] thus proposed that physical tortuosities measured under reaction conditions differ from those measured under diffusion-only conditions. Experimentally-measured tortuosities may additionally include molecule-specific contributions from different interactions with the solid surface, depending on the molecular probe used to make the measurement [76].

The more recent availability of imaging methods that can produce reconstructions of the void space has enabled the determination of geometric measures of tortuosity via image analysis algorithms [67]. Fu et al. [67] reviewed various algorithms, such as Dijkstra’s algorithm and the fast marching algorithm, which were used to determine geometric measures of tortuosity from images. These authors highlighted some shortcomings of such algorithms, such as insensitivity to constrictions in pore cross-section that can restrict diffusive fluxes, which arise because many algorithms reduce the void space to a simple, skeletal graph, thereby losing information about the pore cross-section. Even the geometric tortuosity measures can have a directional element since the shortest path might be obtained only in a particular direction. Hence, overall, geometric measures of tortuosity may be incommensurable with mass transport measures. However, images may be used to perform simulations of mass transport processes within the model void space to obtain predictions of mass transport measures of tortuosity (see below). Overall, when tortuosity is being measured or predicted, then the boundary conditions of the mass transfer process (characteristic length scale and direction) and molecule also need to be specified.

## 3. Representations of the Void Space

### 3.1. Imaging

Tomographic and other 3D imaging methods only provide models of the void space. This is because so-called ‘direct’ imaging methods are not actually direct visualisations of the void space since, as described above, 3D tomographic images are only produced after the application of some sort of reconstruction algorithm to a series of 2D projections or similar [3,13]. For example, 3D X-ray or electron tomography uses a backprojection reconstruction algorithm to determine the most likely 3D object that would be responsible for the set of 2D ‘shadow pictures’ acquired in the experiment by firing the probe radiation beam through the sample from many different directions [77]. The resolution achieved depends upon the number of such projections obtained and from which angles. Dual-beam microscopy requires a succession of 2D slice images of the porous solid to be re-assembled into a 3D block [78]. These 2D slices are not infinitesimally thin but necessarily have a finite thickness, which means some information may be missing for thick slices.

Once an image is acquired, it may contain artefacts and noise that obscure the details. Hence, subsequent image processing is a key step in deriving an accurate model of the void space [29,79]. While many new image-processing algorithms were introduced, including those based upon artificial intelligence, image processing still involves a significant subjective (human) element, and the techniques involved were reviewed in detail, especially for CXT, by Guibert et al. [79]. The noise in an image can be removed to some extent via the application of a filter, such as a random or Gaussian filter [29,79]. Tomographic images can also contain so-called artefacts that arise from effects such as refractions/scattering and beam hardening (for X-rays). Dual-beam microscopy images often contain offset errors, where the 2D slice images do not quite align to form a regular block. These errors are more systematic and require a more specific image-processing procedure to lessen or remove them [29,79]. In order to convert a pore-scale greyscale image into a void space model, it is necessary to segment the image into clear solid and void regions. The division of the image into different phases (solid and void) requires a segmentation algorithm to decide upon the classification of each image pixel. However, this step also still contains many subjective elements [29,79]. The segmentation process can involve making assumptions about the geometry of pores to aid in the identification of the boundary where image pixel intensities are insufficient alone.

The issues with the segmentation of the void space are exemplified by the recent attempt to compare the specific surface area obtained using a standard BET analysis of gas adsorption data and an analysis of electron tomography images for porous titania materials [80]. Initially, a rather poor agreement was obtained between the specific surface areas from nitrogen adsorption and tomography (e.g., 22.5 and 79.1 m^2^g^−1^, respectively), until the accessible pore volume in the tomographic images was identified using impregnated silver crystallites as a tracer for the surface area to count towards the total. However, Yoshida et al. [80] did not make it clear why an image analysis algorithm could not identify externally accessible porosity, which would correspond to the regions probed by nitrogen, in their images.

More recently, it was suggested that higher-resolution electron tomography (ET) can be used to improve the segmentation of lower-resolution CXT images [81]. Prior knowledge of the pore sizes, pore wall borders, and porosity from ET was used as the training dataset for a machine learning algorithm to improve the segmentation of the CXT images. This helped to improve the values of the pore volume from CXT, which otherwise tend to overestimate the actual value due to halo artefacts, which affect the image contrast at interfaces [81].

Another key issue with imaging approaches is achieving a sufficient sample of the void space so that it is properly representative of the whole [29]. It is possible to measure whether some key void space parameter, such as the local average porosity, reaches a constant value over length scales within the field of view of the image so that the image can be said to be a representative volume. For example, the porosity can be averaged over boxes of ever-increasing side length, and the average obtained for each successive box is monitored until it becomes, more or less, a constant value.

Alternatively, the pore sizes obtained from 3D CXT, of a small elemental volume, can be compared with those from, say, mercury porosimetry data for many pellets from the same batch [74]. If there is a good agreement, then the imaging data may be representative of the batch of pellets as a whole, and the pore-shielding effects that are common in mercury porosimetry are not significant [74]. Yamada et al. [74] imaged a particular batch of bimodal pore-sized, alumina catalyst support pellets with electron tomography (3D-TEM) for a sample volume of 304 × 304 × 53 nm at a resolution of 7 nm to characterise the mesoporosity (modal pore size ~14 nm) and synchrotron X-ray nano-CT for a sample volume of 20^3^ μm at a resolution of 35 nm to characterise the macroporosity (modal pore size ~461 nm). These respective imaged volumes were used as the basis of a hierarchical model of the porous solid at the corresponding length scales. First, diffusion was simulated under a concentration gradient in the void space of the lower-length-scale model, while the solid was considered impermeable. This was converted into an effective diffusivity for use as the continuum transport parameter for the ‘solid’ phase in the larger-length-scale image model. Diffusion was then simulated in the larger-length-scale model with transport permitted through both the macroporosity and the permeable solid phase. The simulations were found to give rise to good predictions of the overall effective diffusivity for the alumina pellets measured using a Wicke–Kallenbach cell [74]. However, it was also noted that, for this particular alumina, the much simpler random pore model of Wakao and Smith [82], with the parameters of the model measured using mercury porosimetry, also gave rise to good predictions of diffusivity. These findings suggest this material was relatively homogeneous, and thus, perhaps, not as complex as many other disordered materials. Hence, the imaged volumes were statistically representative of their respective length scales.

However, while for some composite materials, such as some types of rocks, a finite (often quite small) set of classes of mineral grain types can be identified and sampled via high-resolution imaging, for many industrial materials, such as catalyst pellets, no such discretised and finite classification of composite constituents can be achieved [83]. This would necessitate much more extensive sampling of nanoscale void space parameters than is currently practicable.

There are also certain pore-to-pore co-operative physical effects, such as advanced condensation (also known as the ‘cascade effect’) or advanced melting [3,46,84,85], that occur in disordered materials that are not amenable to modelling with structural models that are constructed using most protocols for multimodal, multiscale imaging via CXT and EM. This is because the operation of the cooperative effect depends upon the particular macroscopic spatial juxtaposition of micro- and/or nanoscopic properties of the void space, such as the pore size and connectivity. Most multimodal protocols only involve limited sampling of the nanoscopic properties of the void space at only a few specific locations and then generalising to the remaining solid. However, macroscopically heterogeneous materials, such as many amorphous catalyst supports, possess long-range correlations in local average porosity, pore size, and pore connectivity that are spatially distributed in non-random ways at the larger scale still [83]. The correlation length of the heterogeneity may even exceed the overall size of the catalyst pellet such that the whole pellet is the representative volume required [83]. The variations in these local properties are also more continuous, in contrast to the more discrete variability found in the composite of different types of mineral grains of many rocks, such as shales, where changes in void space parameters arise discontinuously at grain boundaries.

Many studies of catalytic materials using images as the basis of the structural representation, sometimes combined with digital reconstruction, for predicting mass transport are, thus, only performed on various thin films (such as washcoat layers on monolith channel walls or fuel cell layers), where the set of structural variability is similarly limited, as for specific mineral grains within rocks due to the relatively small size (compared with pellets) of the region of interest (~thicknesses of microns only) [86,87,88]. However, physical effects involving long-range correlations in nanoscale properties can be studied using imaging modalities that are capable of measuring such features of the void space, such as magnetic resonance imaging utilising pre-conditioning with relaxometry or diffusometry pulse sequences [24,34,36]. NMR relaxation time-weighted images were used to study the long-range, cooperative adsorption effect known as advanced condensation (or advanced adsorption) [84].

### 3.2. Pore Models

For reasons that are subsequently laid out below, it is asserted here from the start that all descriptions of the void space of disordered porous solids, even if obtained from supposedly direct imaging methods, are, in fact, only models of a more complex reality. This is because in all cases considered here, some details of the original material are left out of the description, and thus, it is necessarily a simplification, as the term ‘model’ suggests. This simplification may be due to the inherent limitations of the structural characterisation technique used and/or a purposeful idealisation that is intending a model that is not ‘maximally realistic’ [89]. The idealisation process and resulting models can be classified into three basic types based upon the justification for the simplification in the light of the intended use of the model, namely, Galilean, minimalist, and multiple-models idealisation [89]. All three types of idealisation are used to develop models for porous solids and will be discussed in more detail below.

**Figure 2:**
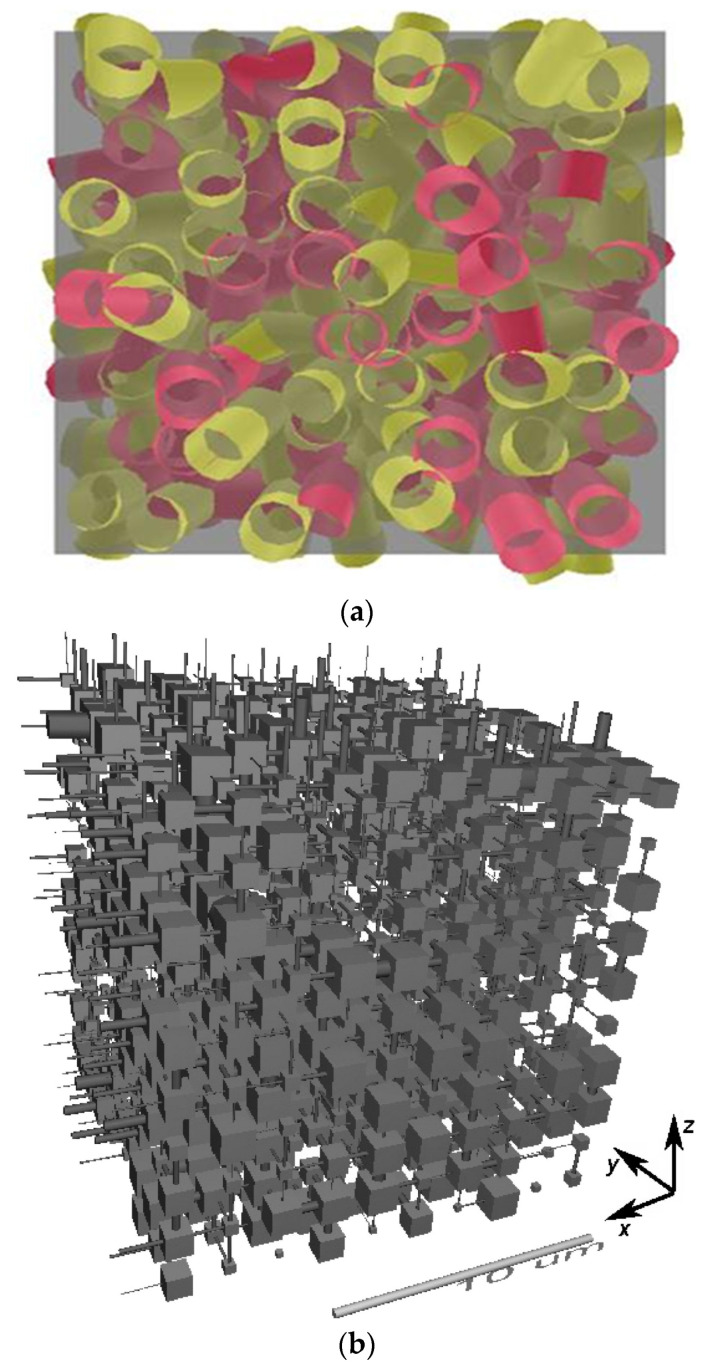
Examples of (**a**) a random pore bond network [106] (Copyright 2017, with permission from Elsevier), and (**b**) a pore neck and pore body network (Pore-Cor), reprinted from [96] (Copyright 2008, with permission from Elsevier).

Galilean idealisation introduces omissions from, or distortions of, the structure of the original material with the aim of achieving sufficient simplification that the description thereby obtained is then mathematically or computationally tractable, both for interrogating the description of the structure itself and using it to make predictions of processes occurring within [89]. As mathematical and computational techniques have become more sophisticated over time, the level of simplification required in Galilean idealisation has diminished. For example, random pore bond networks (see Figure 2a) that are used in modelling catalysts have grown from small (10 × 10), two-dimensional, rectangular grids [17,90] to much larger three-dimensional (50 × 50 × 50 cubic lattice [91]) and even hierarchical lattices [92]. Network models also come in several geometrical variants, including pore neck and pore body networks, such as the Pore-Cor model (see Figure 2b [93,94,95,96]) with cylindrical necks and cubic pore bodies, or with spherical pore bodies [97,98,99,100,101,102]. The spatial distribution of pore sizes within the network models can be completely random or it can be correlated in some way [93,96,100,101].

The level of simplification of disordered void spaces achieved with pore bond network models can be such that it is then possible to conduct a full simulation of complex, coupled mass transport and reaction processes for the pellet scale, including multiple modes of mass transport. These simulations can make a priori predictions of extrinsic reaction rates that can then be compared with an experiment to validate the model, though very few such studies have been performed [103,104,105]. Rieckmann and Keil [103] measured the pore size distribution for the bimodal, meso-/macroporous silica–alumina support for a palladium catalyst using gas sorption and mercury porosimetry. They also obtained the mesopore network connectivity from a percolation analysis of the gas sorption data, as proposed by Seaton [52]. Subsequently, these data were used to construct a three-dimensional, random, cubic network of cylindrical pores. Simulations were then conducted on the model of the coupled diffusion and reaction processes involved in the selective hydrogenation of 1,2-dichloropropane to propane and hydrochloric acid in a single-pellet reactor.

The Galilean idealisation level of pore network models also permits entirely theoretical studies of how different arrangements of pore sizes can potentially impact catalyst activity and selectivity. Different void space geometries can be constructed on a computer, and then their performance for particular reactions can be tested [107,108]. These structures may be based upon existing catalyst support materials that have not yet been tested experimentally for a given reaction or completely hypothetical materials that are yet to be fabricated. The simulations can include the impact of deposition of liquids, as in Fischer-Tropsch gas-to-liquid reactions [75,109] and coke deposits are common in many reactions involving acid catalysts and hydrocarbons [12,110,111]. The models can then be used to optimise pore structures for coking resistance [112,113]. Other Galilean-idealisation-level models for the pore structure of pellets were used to study catalyst activity and selectivity. Various types of lattice-based, fractal cluster models, such as diffusion-limited aggregates (DLAs) and cluster–cluster aggregates (CCAs), for which the construction algorithms are thought to mimic precipitation or gelation processes during the fabrication of supports, were used to model how differential accessibility of active sites can impact catalyst activity and selectivity [114,115,116,117]. For catalyst materials with a relatively limited cross-section, such as monolith washcoats and other thin ceramic films, statistical reconstructions or computer reconstruction of material fabrication methods, such as powder consolidation or slurry flow, can be used to construct reasonable representational models on which subsequent simulations of catalytic reactions were performed, and successfully predicted observed catalyst activities and selectivities [86,118,119]. However, even thin-film materials can also have more multiscale heterogeneity in the pore structure, such as macroscopic/porous cracks that originate from the drying step that are harder to predict a priori, but can also be incorporated into structural representations for modelling coupled diffusion and reaction using CXT images to map the cracks [86]. These models reach the limits of Galilean idealisation as more and more degrees of heterogeneity over increasing length scales (as for a full monolith block, rather than just a single wall section) are needed to be incorporated.

Maps obtained from imaging of the macroscopic spatial variation in local average porosity and pore size of real materials can be used as the basis for the construction of heterogeneous but simplified, site- or bond-type, lattice-based models, where the image data supply the void space descriptors for the model units corresponding to individual voxels [32,120]. These models are particularly appropriate for materials where there are local (<10 μm) correlations in parameters, such as the mesopore size, but these vary over larger length scales. Such model types were used to predict the entrapment of non-wetting fluids [32], as well as mass transport, such as steady-state and transient diffusion [120].

Further, the simplification process can be reversed in so-called ‘de-idealisation’, wherein extra complexity is added back to a simplified model, such as the incorporation of (fractally) rough walls to previously smooth-walled, regular cylindrical pore bonds in network models [121]. However, as discussed below, many industrial materials have a degree of structural complexity that remains beyond the scope of present computing power to completely obviate the need for simplification.

Models formed via minimalist idealisation are only of the degree of complexity necessary to include the particular causal factors that give rise to a phenomenon of interest that occurs within the porous solid [89]. Hence, only those features of the void space structure that ‘make a difference to the occurrence or essential character of the phenomenon in question’ are included [89]. For example, as discussed below, some aspects of a given porous solid do not contribute to controlling the observed flow pattern within the void space and can be reasonably neglected from the model.

**Figure 3:**
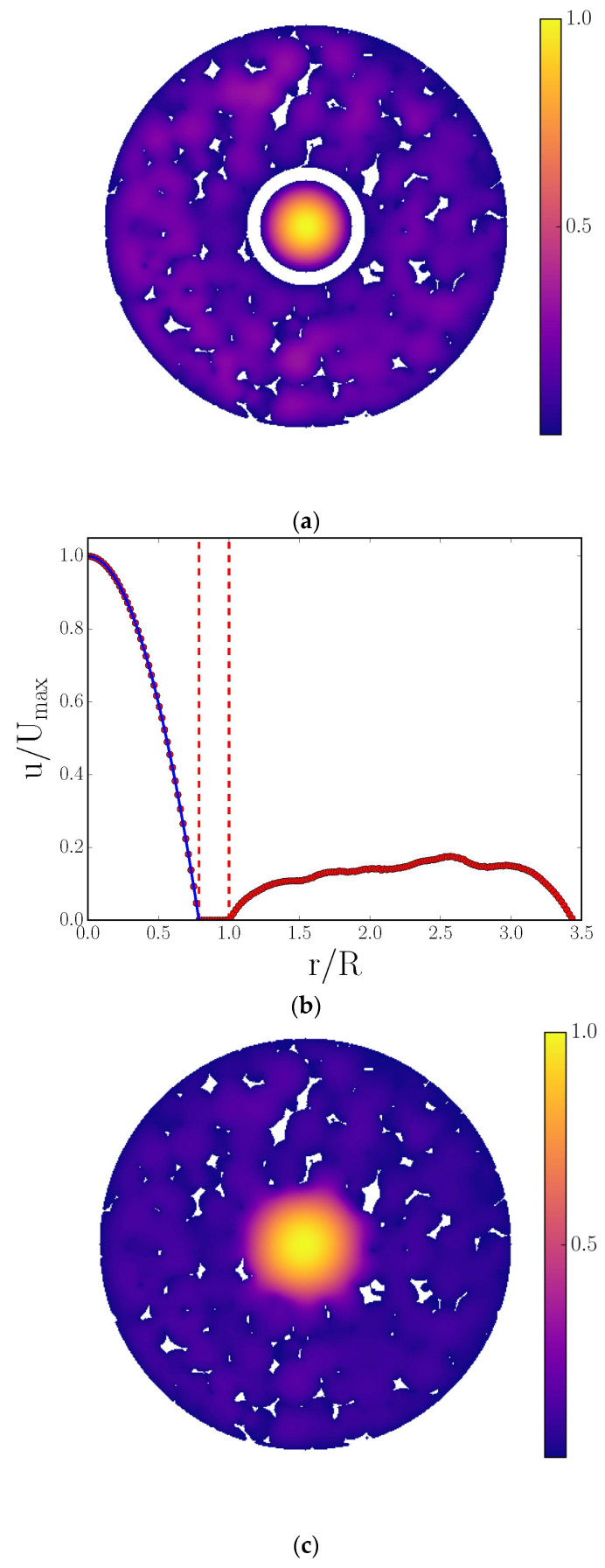

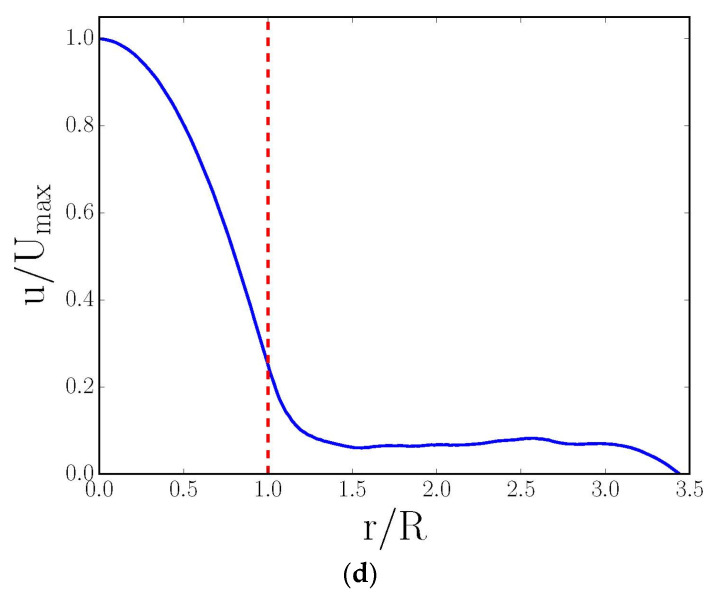
(**a**,**c**) Two-dimensional radial cross-sectional, LB-simulated, normalized velocity maps derived from simulations on CXT images of cylindrical foam monoliths; (**b**,**d**) corresponding normalized azimuthally averaged radial velocity profiles. The data in (**a**,**b**) are in the absence, while (**c**,**d**) are in the presence, of the fluid momentum transport across the interface between a central cylindrical channel and foam walls (which was achieved by inserting a barrier (indicated by white ring) in the former). The blue line in (**b**) represents the analytical solution of the velocity profile for Poiseuille flow in a cylindrical channel with a solid wall (located between the vertical dashed red lines). The single, vertical, dashed red line in (**d**) represents the interface of the cylindrical channel and porous wall. The irregular white shapes in (**a**,**c**) are sections through polymer foam struts. Reproduced under Creative Commons Attribution 4.0 International [28].

**Figure 4:**
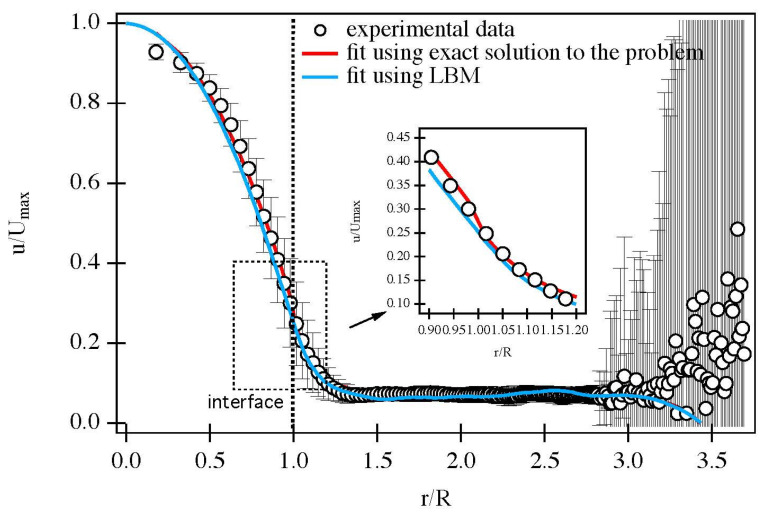
Comparison of an experimental radial velocity profile, measured via MRI, in the presence of fluid momentum transport across the permeable interface between the central channel and porous foam walls at Q = 1.4 cm^3^/s (open symbols) with a formulation of analytical solution due to Kuznetzov [124] (red) and LB simulation (blue). Velocity fields were temporally averaged. The inset shows a zoomed-in comparison of the boundary behaviour of the analytical solution to the problem and LBM modelling. Note a better match to the experiment at the channel boundary with the porous walls for the LB simulation. Reproduced under Creative Commons Attribution 4.0 International [28].

An example of a minimalist model is the so-called ‘brute force’ method for the prediction of transport properties of porous solids uses tomographic images as supposedly full, direct representations of the void space on which to perform mass transport calculations to predict transport parameters, such as diffusivity, tortuosity, and permeability. However, due to the fundamental limitations on the imaging resolution and field of view, even in the most favourable circumstances, the void space representation is still really merely a model. For example, foam-type materials are often used to make highly macroporous ceramic supports for extremely diffusion-limited and/or heat-transfer-limited catalytic systems [83] and are often created initially using polymer-based foams as templates [122,123]. Yeetsorn et al. [123] suggested that the cell architectures of polyurethane (PU) and polyvinyl alcohol foams are attractive for catalyst support preparation due to their high porosities of 98–99% and typical cell pore sizes in the range of 50–200 μm. The cell pores, which are formed by the polymer struts, are thus typically well within the resolution possible with CXT. The voxels of CXT images can then be used for creating the grids for lattice Boltzmann (LB) simulations of mass transport [28]. Such LB simulations on CXT images of PU foams, with cylindrical channels drilled through them (as seen in Figure 3), were found to be better predictive (see Figure 4) of the flow field within the foam, as measured using MRI techniques, compared with conventional analytical solutions [28,124]. The PU foam segment imaged with the CXT was the same segment used in MRI flow measurements, and thus, the FOV was complete in both cases.

However, even the CXT-image-derived grid in Figure 3 is actually only a limited model of the complete void space for the PU foam system. This is because the polymer foam struts (white irregular regions in Figure 3a,c) themselves were also porous, meaning that the system studied was actually a three-level hierarchical porous solid, but the intra-strut pores were below the resolution of the CXT, and thus, ignored in the LB simulation. Although, the comparison with the experiment given in Figure 4 suggests this, apparently, did not detrimentally affect the predictions of the flow field; this was only because fluid exchange with the intra-strut pores was extremely slow on the characteristic timescales of even the flow within the foam cell pores, let alone the wide cylindrical channel. Hence, while this PU foam system is of the type that is the most favourable for the ‘brute force’ approach using imaging, it is still not in complete correspondence with the real system and involves some idealisation. Further, in ‘brute-force’ or direct-image-based, simulations of mass transport, Botha and Sheppard [125] suggested that the minimum required resolution across a pore neck to represent a flow pathway is at least four open voxels such that the appropriate boundary conditions can be represented at the solid–fluid interface.

Minimalist models are especially necessary for systems with high degrees of multiscale complexity since an exhaustive approach would be impossible with current computing capabilities. For some catalyst systems, multimodal imaging suggests that the pellet structure is highly heterogeneous over a broad range of length scales such that it would not be possible to comprehensively characterise enough of the pellet to be representative, and a hypothetical exhaustive dataset would be too large to manipulate (to predict diffusion and reaction) at present, even with current supercomputers [83,126]. In such cases, imaging can still help to inform how much more simplified models of the void space manage to work in terms of being predictive of mass transport properties, and thereby, indicate the limitation on the restricted model’s useful capabilities [3,126]. However, it is not always clear why minimalist models may actually work, and thus, confidence in them may be undermined. However, in cases where imaging may not be able to characterise the whole void space, it can show how the alternative minimalist models achieve their successes.

**Figure 5:**
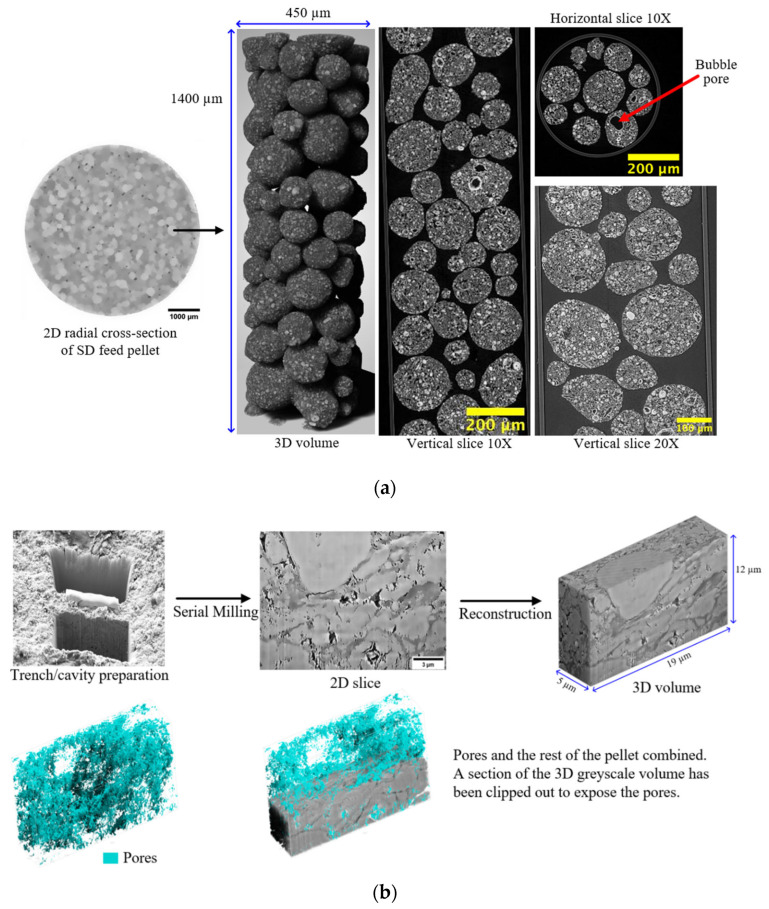
Multimodal imaging of a methanol synthesis catalyst pellet (as seen in Figure 1). (**a**) Two-dimensional radial cross-sections and 3D reconstruction of a high-resolution CXT image of a spray-dried (SD) feed particle that was used to make the SD feed pellet. Furthermore, shown on the left side of the figure, for comparison purposes, is a low-resolution image of a whole SD feed pellet with an arrow indicating a corresponding individual constituent feed particle. (**b**) Two- and three-dimensional reconstructed grayscale FIB-SEM images and the segmentation result for a fresh spray-dried pellet. Furthermore, shown in the figure is the trench/cavity site. The scale bar corresponds to 3 μm. The denser spheroidal region is evident from the void amidst the scatter of (blue) macropores picked out by image segmentation. Reprinted from Mousa et al. [126] under a Creative Commons CC-BY Licence.

For example, multimodal imaging of methanol synthesis catalyst pellets involving CXT with different resolutions and FIB-SEM, as seen in Figure 5, showed that they possess structural heterogeneities over the length scales of the whole pellet, the pellet feed particles, the feed particle constituent fragments, and the pore scale itself [83,126]. From Figure 5, it can be seen that there is some visual similarity in the internal morphology of the pellet, the spray-dried (SD) feed particle, and the constituent fragment of feed in the FIB-milled slice such that, overall, there is a fractal-like appearance. The pellet is composed of smaller spherical SD feed particles of variable density, as shown by differences in the image’s pixel intensity, which resulted from variations in X-ray absorbance (whiter shades mean higher density). The higher-resolution synchrotron CXT images show that these feed particles are, themselves, composed of still smaller, spheroidal fragments of variable density, some of which are hollow, and thus, consist of central voids (black circles) surrounded by (brighter white) shells. The FIB-SEM images also show that the feed particles contain still-smaller spheroidal solid regions (as sectioned in the upper and front surfaces of the FIB-SEM slice) with a different density to the surrounding matrix. The FIB-SEM slice also shows heterogeneity in the spatial distribution of pores (shown in blue in Figure 5b) of sizes larger than imaging resolution, with a notable absence of these in the said spheroidal region, where a noticeable cavity that is much less filled with blue is seen in the rendering of just the pores. Similar levels of the structural complexity of a fractal character were also found for tabletted pellets made with roll-compacted (RC) feed particles [126]. While the apparent fractal character of the SD and RC feed pellets hints at some potential mathematical compression of the exhibited structural complexity, the degrees of disorder are still enormous.

**Figure 6:**
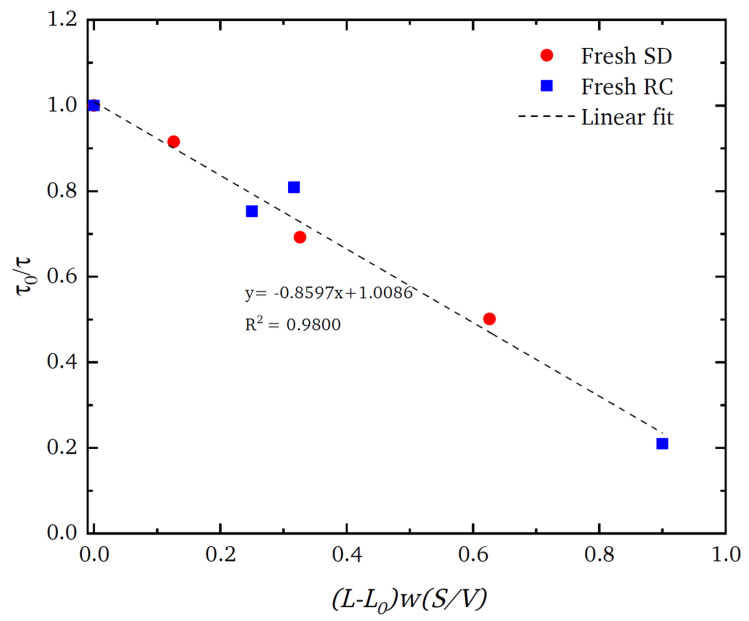
Plot of the ratio of tortuosity τ for nitrogen gas uptake at low pressure before (denoted by subscript 0) and after different levels of mercury entrapment in spray-dried (SD) (red circles) and roll-compacted (RC) (blue squares) feed catalyst pellets against the corresponding change in the apparent lattice size *L*. The parameter *w* is the pellet feed size in each type of pellet, and *S/V* is the external surface-area-to-volume ratio of the finished pellet. Mercury entrapment increased with the increasing value of the variable on the abscissa. Reprinted from Mousa et al. [126] under a Creative Commons CC-BY Licence.

However, despite the extreme levels of structural complexity in the pellets, as is evident in the images in Figure 5, the gross mass transport properties of the material can still be represented by a relatively simple, random, pore bond network model [126] (see Figure 2). The pore bond network was described by two characteristic parameters, namely, the pore bond connectivity *Z* and the overall lattice size *L*. The cylindrical pore bonds were characterised by individual pore diameters and a collective power law exponent for the general relationship between those diameters and pore length. These parameters were all obtained from a percolation analysis of the combined nitrogen gas adsorption and overcondensation desorption isotherm data for the pellets. Controlled modifications were made to the void space of the catalyst pellet via the entrapment of mercury in progressively smaller pore sizes using porosimetry, and then the model parameters were measured for the modified structure. As shown in Figure 6, it was found that the change in the measured tortuosity *τ* for mass uptake into the modified pellet void space relative to that for the original empty pellet (*τ*_0_) is related to the change in the apparent size of the percolation model lattice size (*L* − *L*_0_) following mercury entrapment according to

(2) $$\frac{\tau_{0}}{\tau }=1-\gamma \left(L-L_{0}\right)w{\left(\frac{S}{V}\right)}_{t},$$

where *w* is the pellet feed particle size; *S/V* is the external surface-area-to-volume ratio of the finished pellet; and the ratio of the geometry factors for the feed particles and whole pellet is denoted by *γ*, which is equal to unity if the feed particles and pellet have the same geometrical shape.

Mousa et al. [126] proposed that, even for very complex structures, the critical model parameter for characterising the real pellet macroscopic structure is the lattice size because this parameter is a measure of the prevalence of surface clusters of larger pores. It is these pores that control an invasion percolation process, such as gas desorption or mercury intrusion, as well as the mass transport of gas entering the pellet. The roundedness of the percolation knee in the gas overcondensation curves represents ‘premature’ penetration of the invading vapour phase before the critical pressure required to create a fully sample-spanning, percolating, vapour-filled network is achieved. For a wholly random structure, the probability of generating surface clusters is higher with the greater surface-area-to-volume ratio of smaller lattices simply because a given pore is then more likely to be located on the surface.

**Figure 7:**
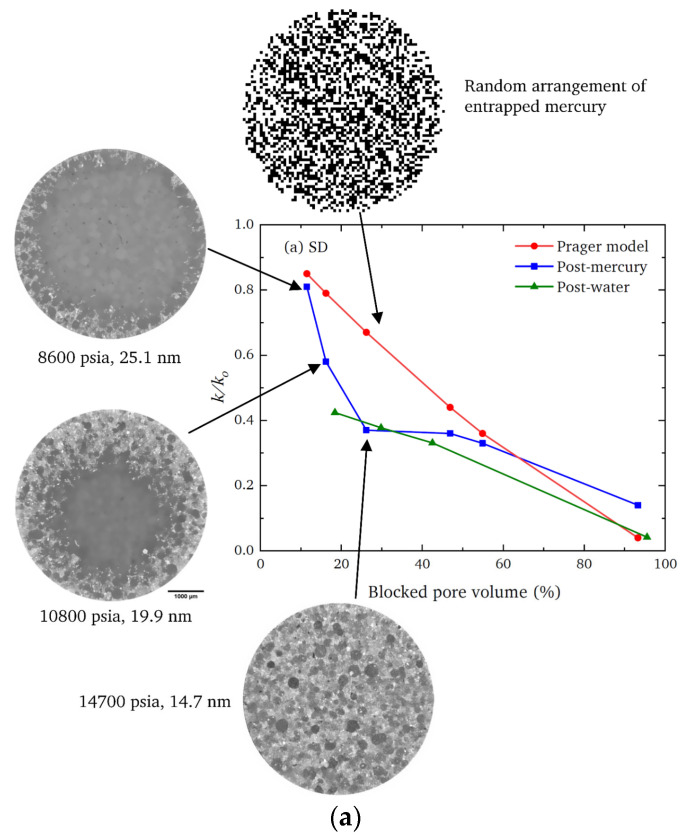

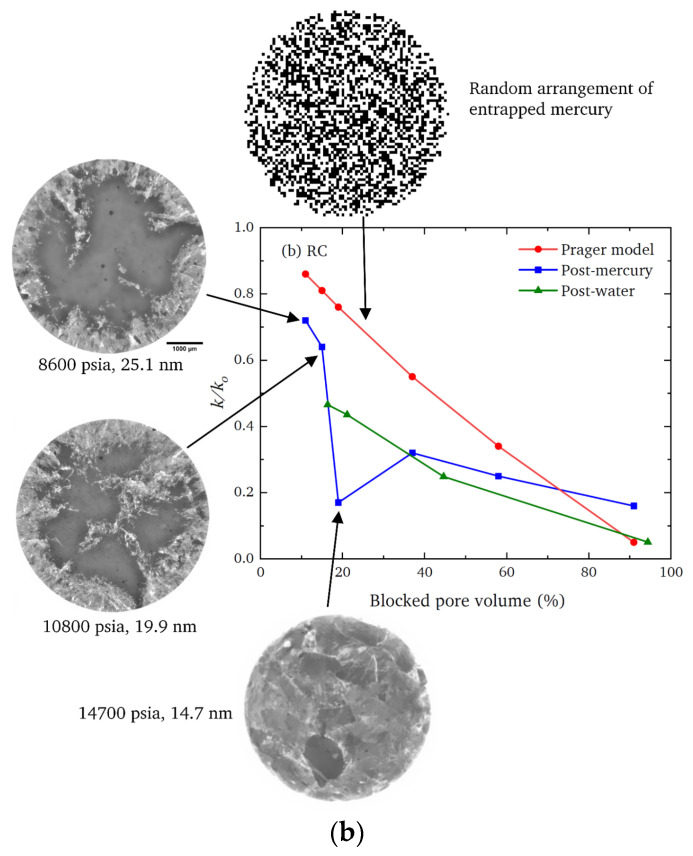
Comparisons of the observed fractional decline in the mass transfer coefficient (*k/k_0_*) following mercury entrapment (blue squares) and water adsorption (green triangles) and that expected for a random arrangement of entrapped pore liquid from the Prager model (as described in [83]) (red circles) for (**a**) SD feed and (**b**) RC feed pellets. The pressures and pore sizes correspond to the ultimate values achieved in the mercury intrusion scanning curves. Reprinted from Mousa et al. [126] under a Creative Commons CC-BY Licence.

If a random lattice (as in Figure 2a) is used to represent a real heterogeneous catalyst, an apparent low model lattice size indicates a higher incidence of surface clusters than for a completely random structure. For example, the ‘shrinking core’ form of the spatial pattern of the progressively advancing mercury penetration front in the CXT images of the SD feed pellets (in Figure 7a) revealed, via a mercury entrapment, the presence of surface clusters of larger pores in this material. This feature of the void space is also registered in the steep rise in apparent model lattice size, as mercury entrapment occluded the surface clusters of larger pores such that they can no longer facilitate early nitrogen desorption. The abstract model lattice size is therefore a proxy for the incidence of surface clusters of larger pores, with apparently smaller lattice sizes corresponding to higher incidence. The presence of surface clusters of larger pores provides easier access and shorter pathways to the interior of the pellet. Consequently, a pellet with an apparently larger lattice size would be associated with higher tortuosity.

The CXT images of the invading mercury front in roll-compacted (RC) feed pellets (shown in Figure 7b) demonstrate that the particular surface clusters in this type of pellet have a very different geometric form to those of the corresponding SD feed pellet (shown in Figure 7a). However, the data for each type of pellet given in Figure 6 demonstrates that the surface cluster structure for each can still be ‘mapped’ onto the surface clusters of an equivalent random pore bond network lattice. The apparent lattice size of the abstract model network is a proxy ‘measure’ of the relative prevalence and penetration depth of the real surface clusters. Hence, while not providing an exhaustive characterisation, the imaging can still indicate how the simple random pore bond network can be predictive of mass transport parameters for even very complex void spaces if it ‘sifts out’ or ‘captures’ the key aspect (in this case, surface clusters) of the void space controlling diffusion rates [126].

Multiple-model idealisation is ‘the practice of building multiple related but incompatible models, each of which makes distinct claims about the nature and causal structure giving rise to a phenomenon’ [89]. This approach is used when a feasible, common, single model cannot explain and/or predict all of the phenomena of interest that arise within a porous solid. In catalytic reactors, there are many physical processes that occur over various different ranges of length scales (as in Figure 1) and time scales, and one modelling approach may not be suitable to include all of these [127]. Where multiple models are needed, it may not be easy to integrate the outputs from each into a grand unified scheme. For example, descriptions of porous media fall into two basic and incompatible types, namely, continuum approaches and discrete representations [128]. In the former, the effects of the void space on processes therein are captured by means of a single, often a-posteriori-adjustable, correction factor. In contrast, discrete approaches take into account the influence of the pore-scale structure on transport parameters by, say, using explicit representations of void space structure. In some cases in catalysis modelling, it may be necessary to model a pore-scale process with mesoscale methods, or even atomistically, in order to capture the necessary degree of complexity of the process. The adsorption of molecules on metallic catalytic centres and specific molecular–surface interaction involved in the chemical reaction itself can generally only be understood using quantum mechanical calculations [127]. The modelling of restructuring of support surfaces and catalytic nanoparticles may require atomistic molecular dynamics approaches. However, detailed modelling of catalyst sites in amorphous materials is problematic due to the diversity of local environments and the lack of structural information for all these environments, while the inhomogeneous distribution of catalytic sites in amorphous materials can significantly affect the overall rates [127]. For atomistic approaches to amorphous materials, there is no general prescription for the placement of peripheral atoms [127]. Further, due to the computing requirements, it is not possible to model mass transport across macroscopically heterogeneous pellets using atomistic methods (e.g., molecular dynamics), and thus, a continuum model may be used to describe the coupled mass transport phenomena. For example, a continuum model might be used to obtain an approximate prediction of the field of concentration of reactants and products across a pellet, which can be used as the input in the first round of a different model for smaller length scales across the pellet. However, the potentially nonlinear feedback between the smaller-scale process, such as adsorption and reaction, and molecular concentration field from mass transport may make integrating the models difficult.

## 4. Outlook

As mentioned above, increasing computer power has meant that ever-larger datasets can be manipulated, meaning that more extensive images or structural models can be developed. The imaging resolution with even lab-based, as well as synchrotron, CXT has steadily been improving. These ongoing developments in hardware technology are expected to continue. For example, studies were recently conducted on ~8 mm diameter porous alumina compacts with nanotomography using a phase-contrast holotomography setup on a synchrotron that was able to image with a pixel size of 25 nm for a field of view of 64 × 64 × 54 μm^3^ [9]. However, it should be noted that a full-sized pellet contains ~10^6^ such sample volumes, and thus, it is still prohibitive to fully image even just a single pellet, never mind a representative sample of several pellets from a heterogeneous batch. Notwithstanding this still-standing sampling issue, high-resolution imaging has enabled the more direct sizing of larger mesopores and the examination of pore connectivity directly, including the identification of relatively larger pore necks. This enabled the finding of unexpected results, such as that the pore size distribution curves remained of similar shape, average size and spread, up to quite long calcination times, and thus, high pellet densities [9].

In order to study ever larger samples with tomography, the beam strength must be increased to enable it to penetrate the sample to produce the projections. However, the potential for increased beam-inflicted damage to samples still limits the beam strength that is possible with X-ray and electron tomography, and thus, the upper size limit of samples. However, there were recent developments in hardware, such as X-ray (and neutron) dark-field imaging using grating interferometry to allow for the quantification of sizes of features below the resolution [129]. Dark-field imaging is based upon the radiation scattering behaviour of the material, and the contrast obtained depends on the unresolved microstructure such that it can obtain pore sizes for void spaces below the resolution of radiation absorption images.

There are also likely to be continued ongoing developments in mathematical techniques and software for data analysis. To overcome the trade-off between the field of view and resolution discussed above and to remove artefacts, artificial intelligence (AI) methods are increasingly being used for image processing [130,131]. Deep learning algorithms are being used to improve super-resolution (SR) reconstruction problems in CXT [130]. SR is used with the aim to reconstruct high-resolution images from multiple low-resolution images, commonly with defects, such that coarse images obtained for large fields of view can be enhanced artificially, and this enhancement is now being done by AI systems [130,131]. It is claimed that AI can produce physically realistic SR images that are predictive of porosity and mass transport properties. The SR reconstructions created from the low-resolution images were validated by comparing various void space metrics against the equivalent for high-resolution images [130]. The types of AI systems utilised include neural networks. Neural networks (NNs) require sufficiently large training datasets to make them predictive. The NNs can then be used to generate representations that are typical of a porous medium to scale-out the model to larger length scales or predict properties for porous materials of a similar type to those used for the training. However, scaled-out models, which represent bigger material volumes, are still unfeasibly large for current computing to be able to conduct direct modelling of mass transport using LB, for example, and thus network models are still needed. However, for some materials, the predictions of mass transport over increasing length scales are reasonably accurate [130]. Further, where generative adversarial networks (GANs) are used to create reconstructions of porous media from low-dimensional latent variables, Huang et al. [131] suggested that the lack of understanding of the synthesis mechanism of the network means that the quantitative relationship between latent variables and the pore structural representation is not clear. This means that GANs are sometimes limited in their ability to distinguish between different morphologies of disordered porous materials.

The development of coupled characterisation and representational approaches for use with multiscale, hierarchical porous systems is also ongoing in order to cope with ever more complex materials. In the homogenisation approach, some averaging law is used to incorporate small-scale physical properties into an upscaled, continuum representation [132]. This statistics-based approach is based upon obtaining correlations between the characteristics of a porous material that are observable in low-resolution images and the high-resolution-image-computed mass transport properties, and then using these correlations to make predictions for new systems [125]. It enables the prediction of transport properties from images where the resolution is too low to permit direct estimation and has some similarities with the AI methods mentioned above. Multiscale percolation systems were used to represent invasion processes, such as the ingress of non-wetting fluids, in hierarchical porous media [133]. An alternative approach used pore bond network models that had separate bonds to represent microporosity in series or parallel with macroporosity. One version of this approach used a method employing SEM images to construct a multiscale pore network model that incorporated spatial and statistical properties of the void space at different resolutions [134,135]. Another upscaling procedure used the random allocation of porosity and transport properties to individual lower-length-scale regions in grids that represent an upper length scale [136]. Many of the methods work best for porous materials with particular characteristics, such as the ways smaller-length-scale properties are distributed across larger-length-scale structures.

## 5. Conclusions

The structural characterisation of so-called amorphous or disordered porous solids still presents an outstanding challenge due to the often complex hierarchy of heterogeneities in void space structure in such materials. Key void space descriptors, such as porosity, pore size, and tortuosity, can now be obtained directly from imaging, but only for limited volumes of the void space. Other, more indirect, techniques allow for the mapping of these parameters for void spaces below the resolution of the image, but for more statistically representative sample volumes. Hence, the complex hierarchy of heterogeneities in the void space of disordered materials can be characterised sufficiently to construct meaningful models of many porous catalysts. The representations of disordered, heterogeneous catalysts are, broadly, of three basic types, which are dependent on the level and purpose of the idealisation of the model.

It was seen that some catalyst pellets have a sufficiently homogeneous void space such that relatively small fields of view are representative of the larger whole, and thus, simulations of mass transport are predictive, along with more idealised random models. However, it was also seen that many other types of catalyst pellets are so heterogeneous that they have hierarchies of complexity over multiple length scales. While it was seen that multimodal imaging can detect this heterogeneity, a full (exhaustive) representation of the void space would be beyond the current computing power. However, it was also seen that more indirect but multiscale porosimetry methods, such as overcondensation and mercury porosimetry, can bridge the many levels in the hierarchy of complexity in one experiment, and can also sift out, or abstract, the key aspects of the void space controlling mass transport. In combination with complementary theoretical modelling approaches, such as percolation analysis, these methods can also extract an underlying commonality of basic form from an otherwise apparent diversity of complex patterns. For example, it was found that both the complex patterns (gasket-like advancing front and the fractal, dendritic branching) of mercury-filled surface clusters of large pores observed in SD and RC feed methanol synthesis catalyst pellets could be mapped onto the simpler surface clusters of random pore bond network models using gas overcondensation data. This type of idealised model captured the key aspect of the void space, namely, the surface clusters of larger pores that controlled mass transport in the modified pore structures, and thus, allowed the model to be predictive of the transport properties. In this case, the complementary imaging data helped to explain why the minimally idealised model was successful.

Due to the multifarious types of void space in disordered porous materials, it is necessary to use some tool, such as the ‘sifting’ or ‘filtering’ approach described above [126], to determine the basic form of the complex hierarchy of heterogeneities in order to select the best strategy for further structural characterisation and suitable model representation. While ways were discussed that aim to link together datasets from various length scales obtained with different imaging modalities, the sampling issue still stands regarding using imaging-only approaches with the most disordered materials. However, multiscale porosimetries, such as mercury porosimetry, and the rarely used gas overcondnesation method permit the knitting together of data from imaging for much bigger sample volumes. The present state of the art thus still requires the continued combination of indirect and more direct characterisation methods.

## Data Availability

Data sharing not applicable.
